# A novel CT image de-noising and fusion based deep learning network to screen for disease (COVID-19)

**DOI:** 10.1038/s41598-023-33614-0

**Published:** 2023-04-23

**Authors:** Sajid Ullah Khan, Imdad Ullah, Najeeb Ullah, Sajid Shah, Mohammed El Affendi, Bumshik Lee

**Affiliations:** 1 Multimedia Information Processing Lab, Department of Information and Communication Engineering, Chosun University, Gwangju, South Korea; 2 Prince Sattam Bin Abdulaziz University, Al-Kharj, Saudi Arabia; 3 Department of Computer Science, University of Engineering &Technology, Mardan, KPK Pakistan; 4 EIAS Data Science Lab, College of Computer and Information Sciences, Prince Sultan University, Riyadh, 11586 Saudi Arabia

**Keywords:** Diseases, Gastroenterology, Medical research, Engineering, Mathematics and computing

## Abstract

A COVID-19, caused by SARS-CoV-2, has been declared a global pandemic by WHO. It first appeared in China at the end of 2019 and quickly spread throughout the world. During the third layer, it became more critical. COVID-19 spread is extremely difficult to control, and a huge number of suspected cases must be screened for a cure as soon as possible. COVID-19 laboratory testing takes time and can result in significant false negatives. To combat COVID-19, reliable, accurate and fast methods are urgently needed. The commonly used Reverse Transcription Polymerase Chain Reaction has a low sensitivity of approximately 60% to 70%, and sometimes even produces negative results. Computer Tomography (CT) has been observed to be a subtle approach to detecting COVID-19, and it may be the best screening method. The scanned image's quality, which is impacted by motion-induced Poisson or Impulse noise, is vital. In order to improve the quality of the acquired image for post segmentation, a novel Impulse and Poisson noise reduction method employing boundary division max/min intensities elimination along with an adaptive window size mechanism is proposed. In the second phase, a number of CNN techniques are explored for detecting COVID-19 from CT images and an Assessment Fusion Based model is proposed to predict the result. The AFM combines the results for cutting-edge CNN architectures and generates a final prediction based on choices. The empirical results demonstrate that our proposed method performs extensively and is extremely useful in actual diagnostic situations.

## Introduction

**Figure 1:**
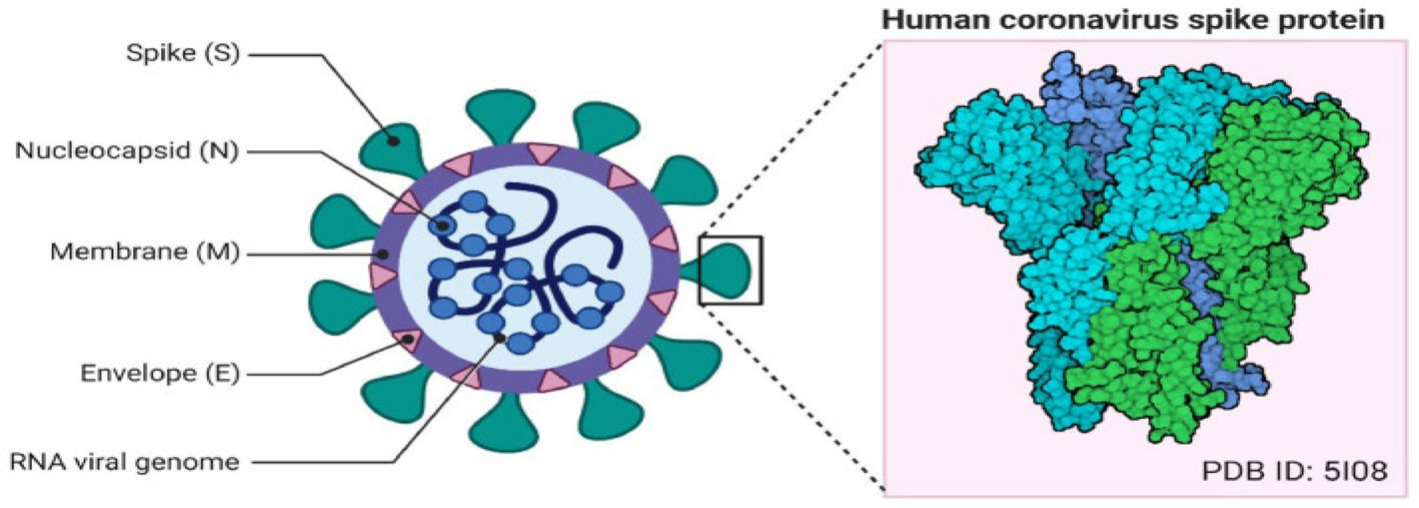
Corona virus structure^3^.

SARS-CoV-2, known as corona virus, causes COVID-19. It is an infectious disease first discovered in China in December 2019^1–3^. World Health Organization (WHO) also declares it as a pandemic. Figure 1 shows its detail structure^3^. This new virus quickly spread throughout the world. Its effect is transmitted to humans through their zoonotic flora. COVID-19's main clinical topographies are cough, sore throat, muscle pain, fever, and shortness of breath^4,5^. Normally, RT-PCR is used for COVID-19 detection. CT and X-ray have also vital roles in early and quick detection of COVID-19^6^. However, RT-PCR has low sensitivity of about 60% -70% and even some times negative results are obtained^7,8^. It is observed that CT is a subtle approach to detecting COVID-19, and it may be a best screening means^9^.

Artificial intelligence and its subsets play a significant role in medicine and have recently expanded their prominence by being used as tool to assist physicians^10–12^. Deep learning techniques are also used with prominent results in many disease detections like skin cancer detection, breast cancer detection, and lung segmentation^13,14^. However, Due to limited resources and radiologists, providing clinicians to each hospital is a difficult task. Consequently, a need of automatic AI or machine learning methods is required to mitigate the issues. It can also be useful in reducing waiting time and test cost by removing RT-PCR kits. However, thorough pre-processing of CT images is necessary to achieve the best results. Poisson or Impulse noise during the acquisition process of these photos could have seriously damaged the image information^15^. To make post-processing tasks like object categorization and segmentation easier, it is essential to recover this lost information. Various filtering algorithms have been proposed to de-blur and to de-noise images in past. Standard Median Filter (SMF) is one of the most often used non-linear filters^16^.

A number of SMF modifications, including Weighted median and Center weighted median (CWM)^17,18^, have been proposed. The most widely used noise adaptive soft-switching median (NASM) was proposed in^19^, which achieved optimal results. However, if the noise density exceeds 50%, the quality of the recovered images degraded significantly. These methods are all non-adaptive and unable to distinguish between edge pixels, uncorrupted pixels, and corrupted pixels. Recent deep learning idea presented in^20–22^ performs well in recovering the images degraded by fixed value Impulse noise. However, its efficiency decreases with the increase in the noise density and in reduction of Poisson noise, which normally exist in CT images. Additionally, most of these methods are non-adaptive and fails while recovering Poisson noise degraded images. In the first phase of this study, layer discrimination with max/min intensities elimination with adaptive filtering window is proposed, which can handle high density Impulse and Poisson noise corrupted CT images. The proposed method has shown superior performance both visually and statistically.

Different deep learning methods are being utilized to detect COVID-19 automatically. To detect COVID-19 in CT scans, a deep learning model employing the COVIDX-Net model that consists of seven CNN models, was developed. This model has higher sensitivity, specificity and can detect COVID-19 with 91.7% accuracy^23^. Reference^24^ shows a deep learning model which obtains 92.4% results in detection of COVID-19. A ResNet50 model was proposed in^25^ which also achieved 98% results as well. All of these trials, nevertheless, took more time to diagnose and didn't produce the best outcomes because of information loss during the acquisition process. There are many studies on detection of COVID-19 that employ machine learning models with CT images^26–29^. A study presented in^30^ proposes two different approaches with two systems each to diagnose tuberculosis from two datasets. In this study, initially, PCA) algorithm was employed to reduce the features’ dimensionality, aiming to extract the deep features. Then, SVM algorithm was used to for classifying features. This hybrid approach achieved an accuracy of 99.2%, a sensitivity of 99.23%, a specificity of 99.41%, and an AUC of 99.78%. Similarly, a study presented in^31^ utilizes different noise reduction techniques and compared the results by calculating qualitative visual inspection and quantitative parameters like Peak Signal-to-Noise Ratio (PSNR), Correlation Coefficient (Cr), and system complexity to determine the optimum denoising algorithm to be applied universally. However, these techniques manipulate all pixels whether they are contaminated by noise or not. An automated deep learning approach from Computed Tomography (CT) scan images to detect COVID-19 is proposed in^32^. In this method anisotropic diffusion techniques are used to de-noised the image and then CNN model is employed to train the dataset. At the end, different models including AlexNet, ResNet50, VGG16 and VGG19 have been evaluated in the experiments. This method worked well and achieved higher accuracy. However, when the images were contaminated with higher noise density, its performance suffered.Similarly, the authors in^33^ used four powerful pre-trained CNN models, VGG16, DenseNet121, ResNet50,and ResNet152, for the COVID-19 CT-scan binary classification task. In this method, a FastAI ResNet framework was designed to automatically find the best architecture using CT images. Additionally, a transfer learning techniques were used to overcome the large training time. This method achieved a higher F1 score of 96%. A deep learning method to detect COVID-19 using chest X-ray images was presented in ^34^. A dataset of 10,040 samples were used in this study. This model has a detection accuracy of 96.43% and a sensitivity of 93.68%. However, its performance dramatically decreases with higher density Poisson noise. A convolution neural networks method used for binary classification pneumonia-based conversion of VGG-19, Inception_V2, and decision tree model was presented in^35^. In this study, X-ray and CT scan images dataset that contains 360 images were used for COVID-19 detection. According to the findings, VGG-19, Inception_V2 and decision tree model illustrate high performance with accuracy of 91% than Inception_V2 (78%) and decision tree (60%) models.

In this paper, a paradigm for automatic COVID-19 screening that is based on assessment fusion is proposed. The effectiveness and efficiency of all baseline models were improved by our proposed model, which utilized the majority voting prediction technique to eliminate the mistakes of individual models. The proposed AFM model only needs chest X-ray images to diagnose COVID-19 in an accurate and speeding way.

The rest of the paper is organized as: The dataset is explained in section "Meterial and methods". section "Proposed method" explains our proposed approach and section "Results and Discussion" presents empirical results and analysis. section "Conclusion" describes conclusion and the specific contributions along with the future directions for improving the efficiency of the proposed work.

## Meterials and methods

**Figure 2:**
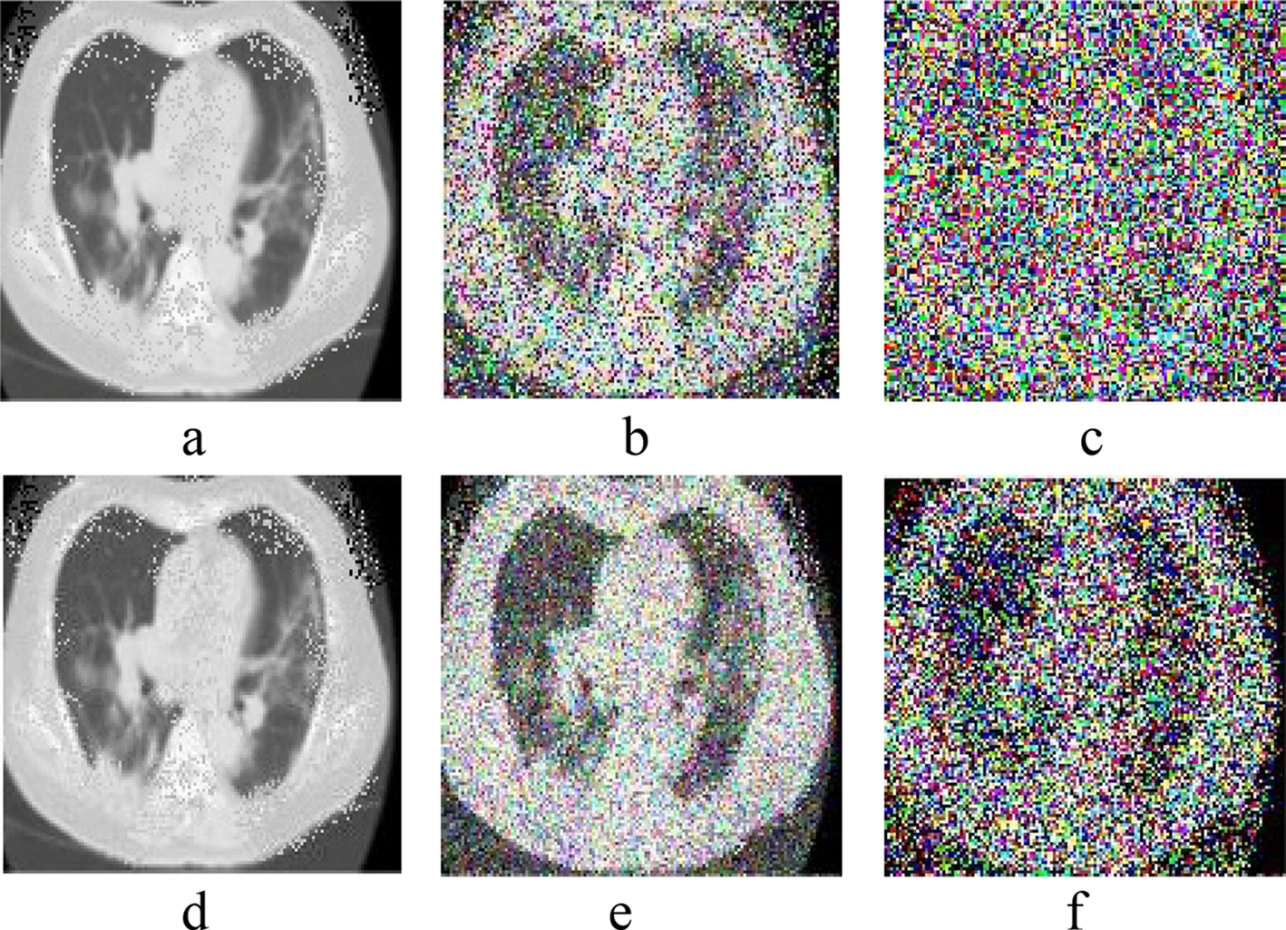
Shows the normal and noisy CT images. (**a**) normal CT image having natural Impulse noise (**b**) Impulse noise corrupted image with 50% noise density (**c**) Impulse noise corrupted image with 80% noise (**d**) Poisson noise corrupted image with 50% noise density (**e**) Poisson noise corrupted image with 80% noise density (**f**) contaminated image with both Impulse and Poisson noise with 80% noise density.

**Figure 3:**
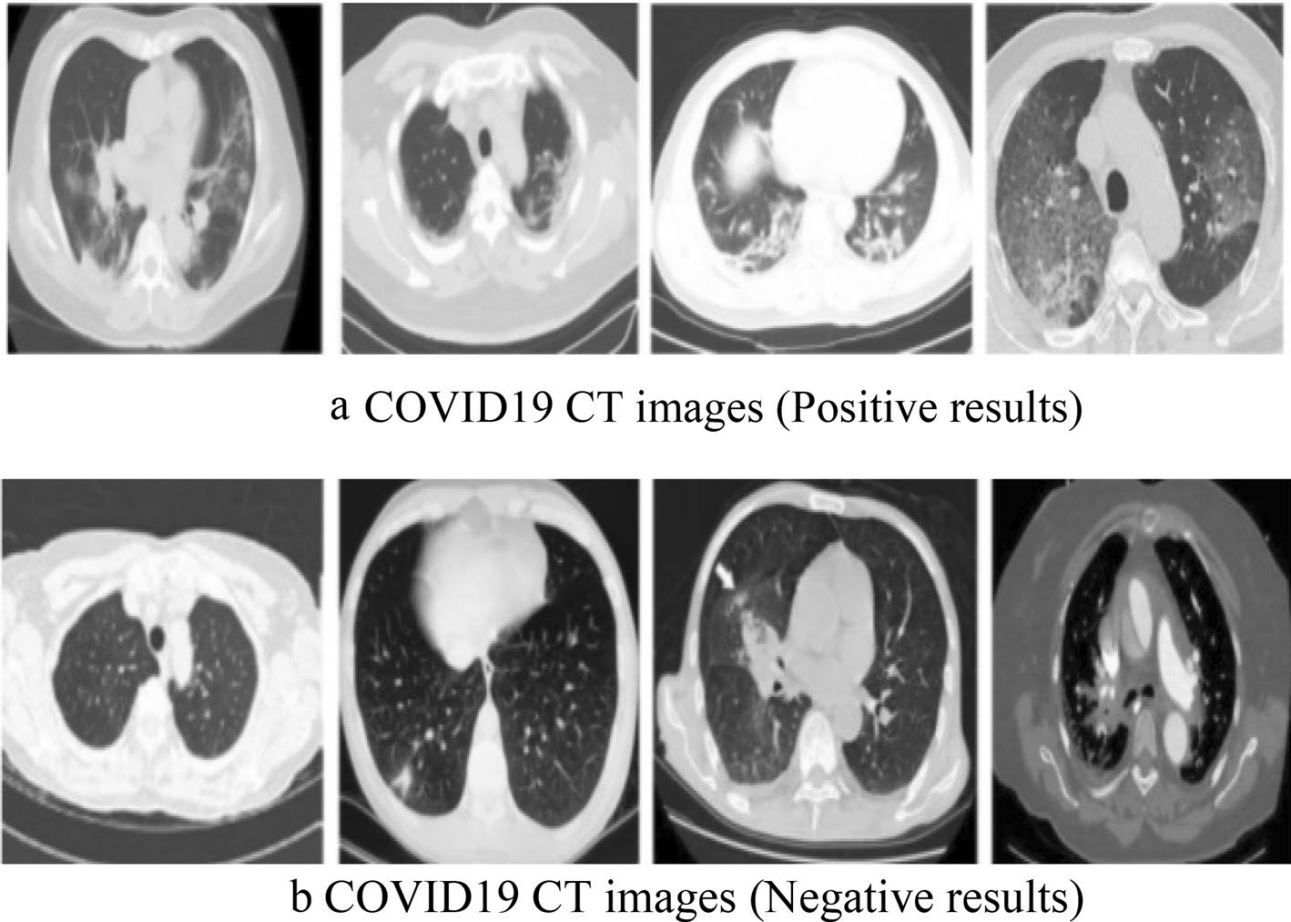
Samples of COVID19 enhanced CT images.

In this study, two types of datasets are used. CT images (dataset) obtain from NIH Clinical Center, Asian Research Hospital, which is publically available at link https://nihcc.app.box.com/v/DeepLesion/folder/51877983116 and from GitHub, available at https://github.com/UCSD-AI4H/COVID-CT. Dataset can also be requested from corresponding author.All methods and experiments in this study were carried out in accordance with relevant guidelines and regulations of the institution (Khalifa Gul Nawaz Hospital, KPK, Pakistan). One dataset consists of 446 images corrupted with Impulse noise and Poisson noise of low and high noise densities. And the second dataset contains 360 COVID positive and 397 COVID negative CT images of different sizes and height. The datasets were split into training and testing parts, 80% used for training and 20% used for testing. Figure 2 shows normal and noisy CT images having different noise densities. Figure 3a,b shows samples of COVID-19 positive and negative de-noised CT images.

Moreover, to improve the throughput and complexity, dataset is converted into JPEG (Joint Photographic Expert Group) format and resize all images to 256*256*3 to keep homogenous characteristics.

### Experiments license

All methods and experiments in this study were carried out in accordance with relevant guidelines and regulations of the institution (Khalifa Gul Nawaz Hospital, KPK, Pakistan).

## Proposed method

The proposed method consists of two phases. The first phase describes the proposed CT image de-noising method while the second phase presents the proposed AFM model in detail.

### CT image de-noising

**Figure 4:**
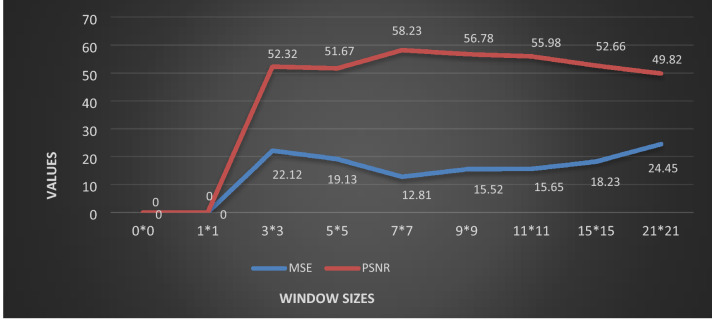
Representation of MSE and PSNR values for various windows.

The first phase explains a novel layer discrimination with max/min intensities eliminations. Adaptive window size for filtering is used to recover CT noisy and blur images degraded by 80% Impulse and Poisson noise density. As a rule of thumb, an empirically determined 7*7 window size is imposed on each pixel to identify the corrupted, uncorrupted and edge pixels globally. It has been observed that large window size improve the speed but add blurriness in the image while small window size can enhance well but increases the steps in computation process. Furthermore, our algorithm checks the noise density in the image and based on the noise density, the window size changes. If noise density increases than 50%, then larger window size of 11 × 11 is adopted. Otherwise, 5 × 5 window is adopted. Figure 4 depicts the Mean Square Error (MSE) and Peak Signal to Noise Ratio (PSNR) values of a 256*256 size CT image that has been recovered using various window sizes. The proposed filtering method consists of two iterations where second iteration being called only under certain conditions. For instance, if one of the observed pixels thought to be noisy, the second iteration will be used for additional validation. To check if the pixel under consideration is uncorrupted, we utilize a window size of 7 × 7. If the pixel is degraded, and it does not come in the middle layer, that is, between L1 and L2, a 3 × 3 window is used in the 2nd iteration to further analyze the pixel based on more focused local statistics to ensure that the pixel is degraded by some noise.

Steps for our proposed layer discrimination with max/min intensities eliminations filtering method are as follow;
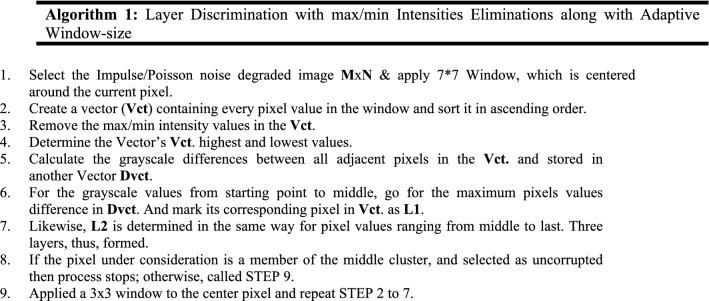


After completing the above process, a Binary Decision Map (BinMp) is created representing corrupted pixels with ‘1 s’ and uncorrupted pixels with ‘0 s’. Binary Map is a separate window uses for calculating the corrupted and uncorrupted pixels. Using the BinMp, all those pixels having grayscale values equivalent to ‘1’ will be replaced by pixels restoration method.

The first pixel of the noisy CT image is often chosen initially, and in a similar manner, the pixel at the same location in BinMp is chosen as well.If it is "0" in BinMp then we move on to the following pixel and stop at the observing pixel since it is uncorrupted. If "1" is found in BinMp, a 3*3 window is imposed on the chosen pixel in the noisy CT image, and vice versa in BinMp. Then, we search BinMp for "0"s and group all items that match into a vector Vct. After that, analyze the vector Vct. and if no element found then move towards next pixel. Else, applied a Wiener filter to the vector Vct. and changed the value of the selected pixel with that Wiener value. Following are the steps for pixel restoration method.
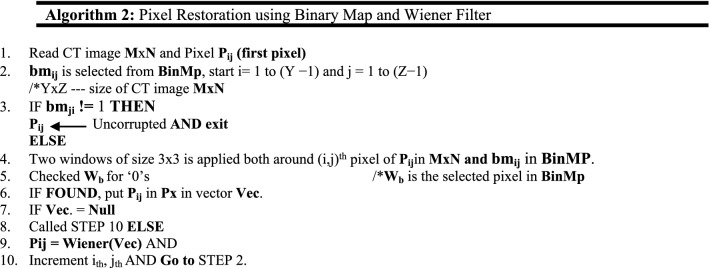


### Assessment based fusion model

**Figure 5:**
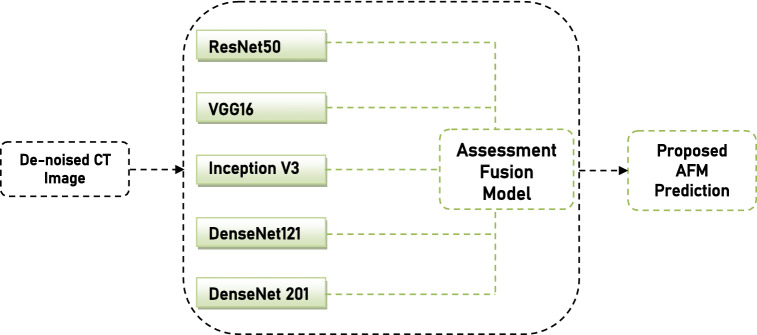
Proposed Assessment Based Fusion Model.

**Figure 6:**
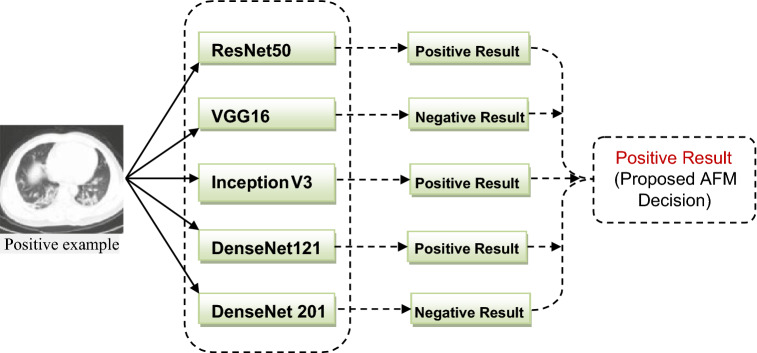
Empirically Assessment of Fusion Based Model.

This part explains the Assessment Fusion based Model (AFM) that is proposed to screen COVID-19 using the enhanced CT images. In this study, different baseline models like VGG16, ResNet50 and DenseNet20 are widely evaluated and the proposed AFM approach is also considered. The concept of our proposed AFM is that it uses the majority voting prediction technique to overcome the errors of individual models to improve the effectiveness and efficiency of all baseline models. The proposed AFM model is graphically represented in Fig. 5, and the empirical evaluation of AFM is shown in Fig. 6.

In the subsections, existing baseline models are briefly discussed.

Below sections briefly explain the state of the art different baseline models used for the detection of COVID-19.

#### ResNet50 architecture

ResNet50 architecture was proposed by^25^ is simple to implement, reduces training time with higher accuracy. However, because of the vanishing gradient, network performance may suffer. This architecture consists of a number of layers having identity connections combinable called residual block. Architecture of ResNet50 includes convolution layers, max pooling layers, and a fully connected layer.

#### VGG 16

VGG16 proposed by^16^ is one of the most popular Deep CNN model. VGG 16 got 93.8% F-Score holding top-5 test accuracy in ImageNet, which is a dataset of over 14 million images belonging to 1000 classes. However, it is very slow to train (the original VGG model was trained on Nvidia Titan GPU for 2–3 weeks)^26^. Additionally, it took up more room on the disk.

#### Inception V3

Inception V3 is a popular GoogleNet and in biomedical field it has remarkable classification performance. Inception model merged multiple size filters into a new filter which decreases the computational complexity and training parameters.

#### DenseNet architecture

DenseNet is the recent findings in neural networks. It is similar to ResNet with minor differences. In this architecture the previous layer is concatenated with future layer.

## Results and discussion

**Figure 7:**
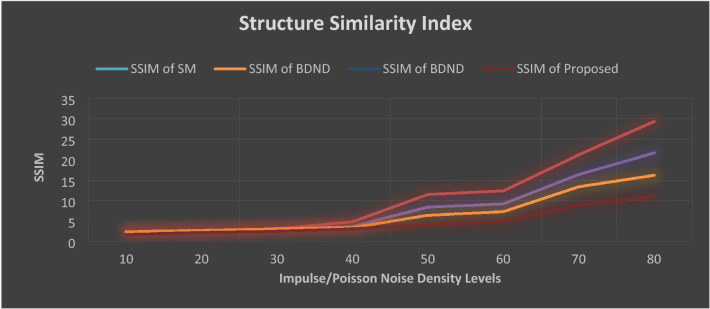
Structure Similarity Index (SSIM) values of all evaluated approaches. Noise density is from 55 to 90%. BDND = boundary discriminative noise detection, SR = square-root.

**Figure 8:**
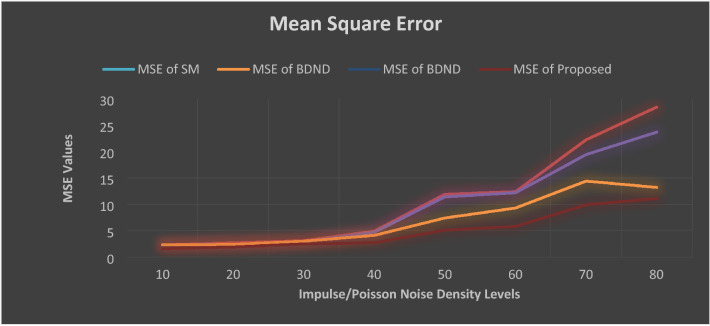
Mean Square Error (MSE) values of all evaluated approaches. Noise density is from 5 to 90%. BDND = boundary discriminative noise detection, SR = square-root.

In comparison to other methods described in^15–22^, empirical findings of our proposed Layer discrimination max/min intensity reduction method shows much higher performance. The SSIM and MSC values of current and hypothetical state-of-the-art methods are shown in Figs. 7 and 8.

Figures 7 and 8 depict that our proposed de-noising algorithm outclass the existing state of the art methods in terms of SSIM and MSE values. For lower noise density such as up to 30%, all methods performed well. However, once the noise density increases, the performance of the existing methods decreases dramatically.

**Figure 9:**
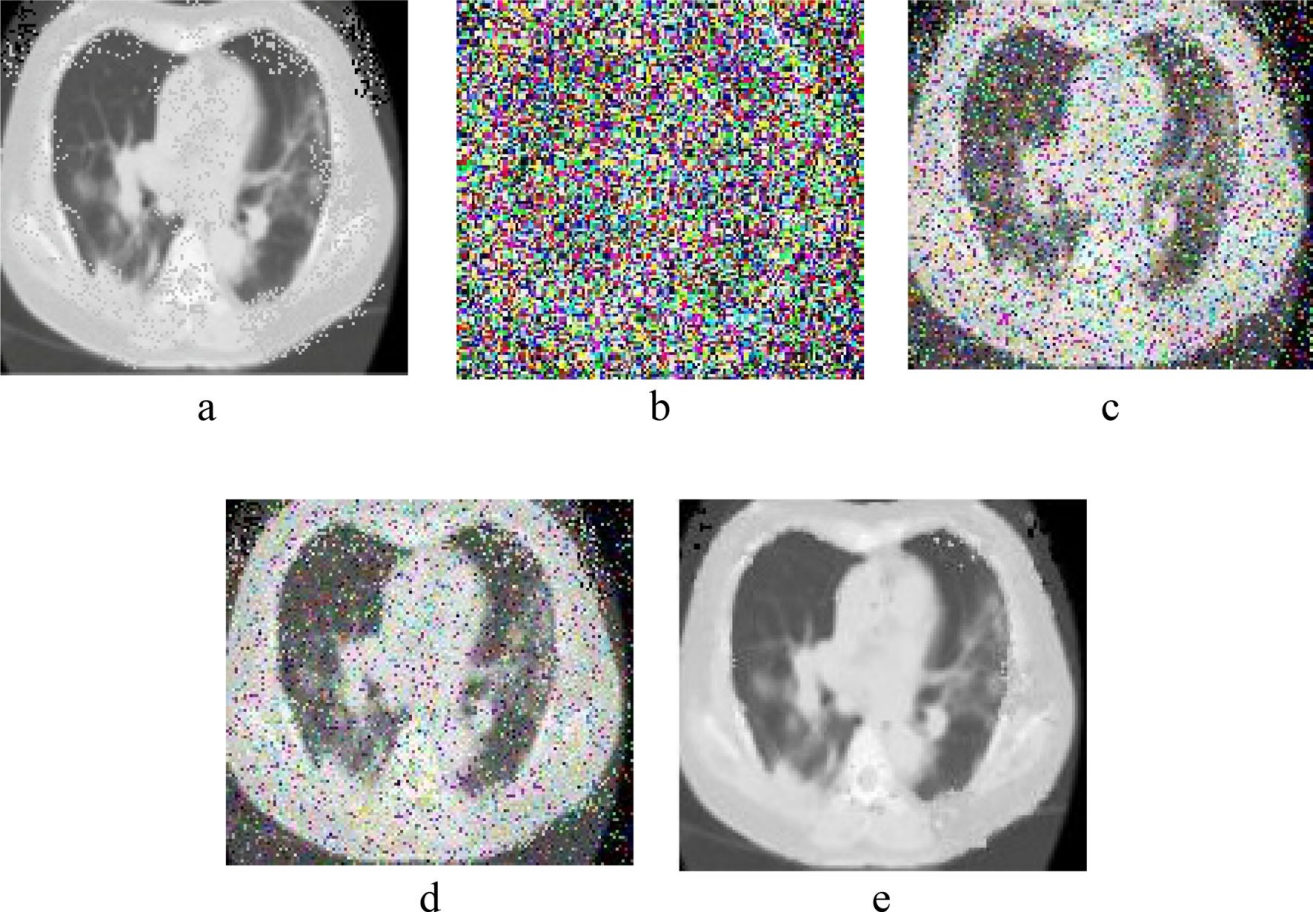
shows the normal, noisy and de-noised CT images. (**a**) Normal CT image having natural Impulse noise (**b**) Impulse noise corrupted image with 80% noise density (**c**) De-noised image with SM (**d**) De-noised image with BDND (**e**) De-noised image with proposed method.

The following Fig. 9 depicts the visually appealing results of the existing and proposed algorithms. On images with up to 80% noise density, SM and BDND perform admirably. However, at noise densities greater than 50%, it completely fails to remove Poisson noise and even Impulse noise.

Figure 9 shows that our proposed filter method outperforms the standard SM and BDND filters. BDND is effective for de-nosing Impulse noise from CT images with a noise density of 50%. However, when the noise density increases, its performance decreases dramatically. The time parameters of the existing state of the art and proposed methods are shown in Table 1.

**Table 1:** Time performance of the SM, BDND and proposed method incorporated on test CT image being corrupted by a total Impulse/Poisson noise density of 80%.

Methods	Types of Noise	Time taken/seconds
Standard SM	Degraded with Impulse noise	21.12
	Degraded with Poisson noise	19.84
	Degraded with Impulse and Poisson noise both	22.87
BDND	Degraded with Impulse noise	16.51
	Degraded with Poisson noise	16.95
	Degraded with Impulse and Poisson noise both	18.21
Our method	Degraded with Impulse noise	11.20
	Degraded with Poisson noise	11.81
	Degraded with Impulse and Poisson noise both	12.51

Similarly, Table 2 outlines the results for 80% Impulse/Poisson noise contaminated images. However, the range of noise in this case having unequal probability. It has been discovered empirically that the measurement of SM and BDND dramatically reduces as the range of noise variation rises.

**Table 2:** Performance of existing and proposed filters with un-equal probability of noise density.

Noise Range		PSNR (dB)		
Min intensity Impulse	Max intensity Impulse	SM	BNDND	Proposed
[0,9]	[246,255]	17.12	19.21	22.27
[0,29]	[226,255]	17.84	19.81	22.86
[0,49]	[206,255]	18.31	20.59	24.41
[0,69]	[186,255]	19.21	21.32	25.22
[0,89]	[166,255]	19.36	21.77	25.34
[0,109]	[146,255]	19.37	21.94	26.21

According to the above-mentioned subjective and objective findings, our filter operates better than the SM and BDND, particularly at larger densities. The proposed method is comparatively quick and easy, as illustrated in Table 3.

**Table 3:** Training and prediction time of all models.

Model	Training Time	Prediction time (for a single sample)
ResNet 50	3129.665412	0.02351945434
Inception V3	2908.542147	0.02540415520
VGG16	2354.320144	0.05641245021
DenseNet 121	3890.556484	0.02754245016
DenseNet 201	5232.552485	0.02584751247
Proposed AFM	1721.325481	0.19840524564

The above-mentioned models are empirically evaluated in the second part of this work using a variety of performance metrics, including F1-Score, specificity, recall, sensitivity, AUC value, and ROC Curve. In COVID prediction and other medical detection systems, these measures are quite helpful. The details of this measure are described in more detail below.

The F1 score is a machine learning assessment metric used to measure the accuracy of a model. It fused the recall and precision scores of a model. How many times a model correctly predicted across the entire dataset is determined by the accuracy metric. Sensitivity and Specificity are two measures of a model's performance. Sensitivity is the proportion of true positives correctly predicted by the model, whereas specificity is the proportion of true negatives correctly predicted by the model. Similarly, Precision is the fraction of relevant instances among the retrieved instances, while recall is the fraction of relevant instances that are retrieved.

1 $$F1\mathrm{Score}= \frac{(TP+FN)}{(TP+TN+FP+FN)}$$

2 $$Sensitivity= \frac{(TP)}{(TP+FN)}$$

3 $$Specificity= \frac{(TN)}{(TN+FP)}$$

4 $$Precsion= \frac{(TP)}{(TP+FP)}$$

5 $$Recall= \frac{2* (Precision*Recall)}{(Precision+Recall)}$$

6 $$Accuracy= \frac{(TP+FN)}{(TP+TN+FP+FN)}$$

**Figure 10:**
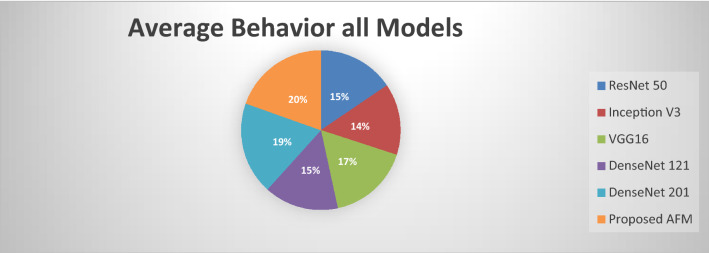
Average behavior of each and every model with 90% of confidence intervals.

The empirical evaluation is repeated 20 times in various random splits. Python 4.0 is used as the front end, and Tensor Flow is used as the backend. 85% of the data in each split is utilized for training, and the remaining 15% is used for testing. To avoid over-fitting and to make an early stop, 10% of the training data is kept as a validation set and the remaining 90% is kept as training data during model training. For model optimization, a Stochastic Gradient Descent Optimizer (SGDO) with learning rates of 0.002 and 0.8 is used. The previously discussed performance measures are used to assess the model's accuracy. Figure 10 represents the average behavior of each model with 90% confidence intervals.

**Figure 11:**
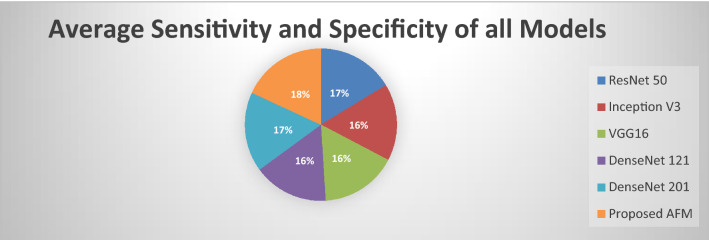
Sensitivity and specificity of all models.

It is empirically determined that our proposed AFM model performs outclass all the existing state of the art models. DenseNet121 has higher accuracy and F1-Score than all other models. Furthermore, the proposed AFM model outperforms others in terms of sensitivity and specificity. The results are shown in Fig. 11. The graph clearly shows that our proposed AFM model significantly improves average specificity.

**Figure 12:**
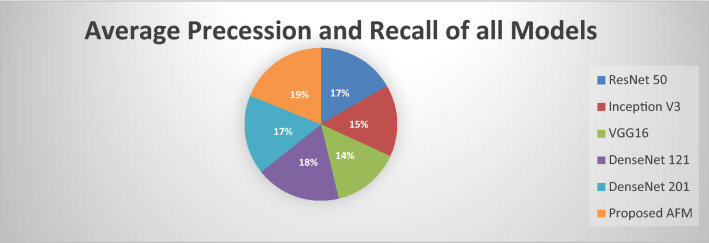
Average Precession and recall of all models.

The outcomes of the Fig. 12 demonstrate that our proposed AFM model outperforms the existing models. Our proposed AFM model's average precession is better than the individual one, indicating that AFM has a much better False Positive Rate.

Moreover, Table 3 presents the prediction and training time of existing models having one sample and the comparison of the proposed AFM.

It has been empirically proven that our proposed filtering technique and Assessment Fusion based Model (AFM) are appropriate in a real-world setting for COVID-19 identification from CT images. The findings demonstrate that the accuracy and computation speed of our suggested filtering approach is satisfactory. Similar to this, the proposed AFM fared better than current cutting-edge models. Additionally, the AMF False Positive Rate has been significantly decreased. The proposed AFM model also decreased the amount of time needed to acquire images that will reduce the patients’ waiting time during scanning.

## Conclusion

This study in its first phase proposed layer discrimination with max/min intensities elimination, a novel, simple, and reasonably accurate Impulse/Poisson noise detection filter for de-noising and de-blurriness of the CT images. Extensive empirical results show that our proposed filter outperforms the existing state-of-the-art standard SM and BDND filters across a wide range of noise densities, achieving higher SSIM, MSE, and PSNR. The second phase involves a detailed evaluation of the several existing models for COVID-19 positive identification from chest CT scans, followed by the proposed Assessment Fusion based Model that integrates the findings of each individual model in order to increase performance. It is empirically determined that our proposed AFM outclasses all existing state of the art models and achieve optimal performance in term of False Positive, sensitivity, specificity and average precisions.

Moreover, the commonly used Reverse Transcription Polymerase Chain Reaction (RT-PCR) has a low sensitivity of approximately 60% to 70%, and sometimes even produces negative results. Our proposed AFM also dramatically reduced the screening time which shows that it can be widely used in real world applications. Furthermore, there is still room for improvement in order to get predictive performance. Some techniques like Image Augmentation and Feature Level Fusion can boost the performance and these ideas are to be explored in future.

## Data Availability

The datasets used during the current study are available at NIH Clinical Center, Asian Research Hospital via linkhttps://nihcc.app.box.com/v/DeepLesion/folder/51877983116 and from Github via https://github.com/UCSD-AI4H/COVID-CT. Data can also be requested from corresponding author.

## References

[1] Wu F, Zhao S, Yu B (2020). A new coronavirus associated with human respiratory disease in China. Nature.

[2] Huang C, Wang Y (2020). Clinical features of patients infected with 2019 novel coronavirus in Wuhan, China. Lancet.

[3] World Health Organization (WHO). Pneumonia of Unknown Cause–China. Emergencies Preparedness, Response, Disease Outbreak News. https://www.who.int/emergencies/disease-outbreak-news/item/2020-DON229 (2021).

[4] Siddiqui MK, Morales-Menendez R, Gupta PK, Iqbal HM, Hussain F, Khatoon K (2020). Correlation between temperature and covid-19 (suspected, confirmed and death) cases based on machine learning analysis. J. Pure Appl. Microbiol..

[5] Singha LT (2020). A review of coronavirus disease-2019 (COVID-19). Indian J. Pediatr..

[6] Zu ZY, Jiang MD, Xu PP, Chen W, Ni QQ, Lu GM, Zhang LJ (2020). Coronavirus disease 2019 (COVID-19): A perspective from China. Radiology.

[7] Kanne JP, Little BP, Chung JH, Elicker BM, Ketai LH (2020). Essentials for radiologists on COVID-19: An update—Radiology scientific expert panel. Radiology.

[8] Xie X, Zhong Z, Zhao W, Zheng C, Wang F, Liu J (2020). Chest CT for typical 2019-nCoV pneumonia: Relationship to negative RT-PCR testing. Radiology.

[9] Lee EY, Ng MY, Khong PL (2020). COVID-19 pneumonia: What has CT taught us?. Lancet Infect. Dis..

[10] Litjens G, Kooi T, Bejnordi BE (2017). A survey on deep learning in medical image analysis. Med. Image Anal..

[11] Ker J, Wang L, Rao J, Lim T (2018). Deep learning applications in medical image analysis. IEEE Access..

[12] Shen D, Wu G, Suk HI (2017). Deep learning in medical image analysis. Annu. Rev. Biomed. Eng..

[13] Hannun AY, Rajpurkar P, Haghpanahi M (2019). Cardiologist-level arrhythmia detection and classification in ambulatory electrocardiograms using a deep neural network. Nat. Med..

[14] Acharya UR, Oh SL, Hagiwara Y (2017). A deep convolutional neural network model to classify heartbeats. Comput. Biol. Med..

[15] Pie-Eng N, Kai-Kuang M (2006). A switching median filter with boundary discriminative noise detection for extremely corrupted image. IEEE Trans. Image Process..

[16] Pitas I, Venetsanopoulos AN (1992). Order statistics in digital image processing. Proc. IEEE..

[17] Brownrigg DRK (1984). The weighted median filter. Commun. ACM..

[18] Ko SJ, Lee YH (1991). Center weighted median filters and their applications to image enhancement. IEEE Trans. Circuits Syst..

[19] Eng HL, Ma KK (2001). Noise adaptive soft-switching median filter. IEEE Trans. Image Process..

[20] Liu, G., Reda, F. A., Shih, K. J., Wang, T.C., Tao, A. & Catanzaro, B. Image inpainting for irregular holes using partial convolutions. In *Proceedings of the European Conf. on Computer Vision (ECCV)*. 85–100 (2018).

[21] Yu, J., Lin, Z., Yang, J., Shen, X., Lu, X. & Huang, T. S. Generative image inpainting with contextual attention. In *Proceedings of the IEEE Conf. on Computer Vision and Pattern Recognition*. 5505–5514 (2018).

[22] Liu, H., Jiang, B., Xiao, Y. & Yang, C. Coherent semantic attention for image inpainting. arXiv preprint arXiv:1905.12384 (2019).

[23] Wang, L. & Wong, A. COVID-Net: A Tailored Deep Convolutional Neural Network Design for Detection of COVID-19 Cases from Chest Radiography Images. arXiv:2003.09871 (2003). 10.1038/s41598-020-76550-z PMC7658227 33177550

[24] Oannis, D. A., & Bessiana, T. COVID-19: Automatic Detection from X-Ray Images Utilizing Transfer Learning with Convolutional Neural Networks. arXiv:2003.11617 (2003). 10.1007/s13246-020-00865-4 PMC7118364 32524445

[25] Narin, A., Kaya, C. &Pamuk, Z. Automatic Detection of Coronavirus Disease (COVID-19) Using X-Ray Images and Deep Convolutional Neural Networks. arXiv:2003.10849. (2003). 10.1007/s10044-021-00984-y PMC8106971 33994847

[26] Song Y, Zheng S, Li L, Zhang X, Huang Z, Chong U (2021). Deep learning enables accurate diagnosis of novel coronavirus (COVID-19) with CT images. medRxiv..

[27] Wang S, Kang B, Ma J, Zeng X, Xiao M, Guo J, Xu B (2021). A deep learning algorithm using CT images to screen for Corona Virus Disease (COVID-19). medRxiv..

[28] Zheng C, Deng X, Fu Q, Zhou Q, Feng J, Ma H, Wang X (2020). Deep learning-based detection for COVID-19 from chest CT using weak label. IEEE Trans. Med. Imaging.

[29] Xu X, Jiang X, Ma C, Du P, Li X, Lv S (2020). Deep Learning System to Screen Coronavirus Disease 2019 Pneumonia. ArXiv..

[30] Fati SM, Senan EM, ElHakim N (2022). Deep and Hybrid Learning Technique for Early Detection of Tuberculosis Based on X-ray Images Using Feature Fusion. Applied Sciences.

[31] El-Shafai W, Mahmoud AA, Ali AM, El-Rabaie EM (2022). Deep cnn model for multimodal medical image denoising. Computers, Materials & Continua.

[32] Uddin KMM, Dey SK, Babu HMH, Mostafiz R, Uddin S, Shoombuatong W, Moni MA (2022). Feature fusion based VGGFusionNet model to detect COVID-19 patients utilizing computed tomography scan images. Sci. Rep..

[33] Yang D, Martinez C, Visuña L, Khandhar H, Bhatt C, Carretero J (2021). Detection and analysis of COVID-19 in medical images using deep learning techniques. Sci. Rep..

[34] Chakraborty S, Murali B, Mitra AK (2022). An efficient deep learning model to detect COVID-19 using chest X-ray images. Int. J. Environ. Res. Public Health.

[35] Dansana D, Kumar R, Bhattacharjee A, Hemanth DJ, Gupta D, Khanna A, Castillo O (2020). Early diagnosis of COVID-19-affected patients based on X-ray and computed tomography images using deep learning algorithm. Soft. Comput..

